# Functional Outcomes of Selective Nerve Root Block in Lumbar Radiculopathy Due to Intervertebral Disc Prolapse

**DOI:** 10.7759/cureus.92460

**Published:** 2025-09-16

**Authors:** Gowtham Gandhi, Prabhu Ethiraj

**Affiliations:** 1 Orthopedic Surgery, Sri Devaraj Urs Academy of Higher Education and Research, Kolar, IND; 2 Orthopedics, Sri Devaraj Urs Medical College, Sri Devaraj Urs Academy Of Higher Education and Research, Kolar, IND

**Keywords:** low back ache, lumbar radiculopathy, numerical rating scale, oswestry disability index, prolapsed intervertebral disc, selective nerve root block, straight leg raise test

## Abstract

Background

Lumbar radiculopathy (LR) due to prolapsed intervertebral disc (PIVD) is a prevalent cause of pain and functional limitation. Selective nerve root block (SNRB) is used both diagnostically and therapeutically; however, pragmatic effectiveness data remain limited.

Objective

This study aims to quantify short‑term changes in pain and disability following fluoroscopy‑guided SNRB for single‑level LR due to PIVD.

Materials and methods

Prospective observational cohort at a secondary‑care hospital. Adults aged 18-60 years with clinicoradiologic concordance for single‑level LR received SNRB. Outcomes were measured using the Numeric Rating Scale (NRS) for pain and the Oswestry Disability Index (ODI) at baseline, immediately post-procedure (day 0), 30 days, and 90 days. The Straight‑Leg Raise Test (SLRT) was assessed at each visit. Paired comparisons evaluated changes from baseline; 30‑ vs. 90‑day differences were also tested.

Results

Thirty‑five patients were included. Mean NRS decreased from 6.40 ± 0.78 at baseline to 3.29 ± 0.57 (day 0), 3.17 ± 0.64 (30 days), and 3.14 ± 0.55 (90 days); all comparisons vs. baseline p = 0.0001; 30 → 90 days p = 0.201. ODI improved from 14.31 ± 3.43 to 7.83 ± 2.26 (day 0), 7.86 ± 2.68 (30 days), and 6.86 ± 2.39 (90 days); all p = 0.0001 vs. baseline. SLRT converted from positive pre‑procedure to negative immediately and at 30 days, with partial recurrence in 4/35 (11.4%) at 90 days.

Conclusions

In this cohort, fluoroscopy‑guided SNRB produced rapid and durable improvements in pain and disability through three months, supporting its use as an intermediate option between initial conservative care and surgery when clinical and imaging findings are concordant. Larger randomized or comparative‑effectiveness studies with longer follow‑up are warranted to assess durability, define predictors of response, and optimize patient selection.

## Introduction

Lumbar radiculopathy (LR) due to a prolapsed intervertebral disc (PIVD) is a frequent cause of pain and disability. Selective nerve root block (SNRB) is used both diagnostically and therapeutically in this context [1]. Clinical series suggest benefit in disc‑related radiculopathy following SNRB [2,3]. Epidemiology and clinical characterization of lumbosacral radiculopathy are well described [4,5], and classic mechanistic descriptions highlight mechanical compression and inflammatory mediators [6,7].

Although many patients improve with conservative care [8,9], some require surgery, and randomized and observational studies have defined criteria and outcomes for operative versus nonoperative pathways [10,11]. Epidural corticosteroid‑based approaches remain part of multimodal management [12]. Diagnostic SNRB may help confirm the symptomatic level when clinicoradiologic findings are discordant [13-15], and therapeutic injections can extend analgesia and improve function in selected patients [16]. Technique overviews and guidance synthesize indications and procedural principles [17,18].

Recent cohorts support the effectiveness of SNRB or transforaminal injections in disc‑related radiculopathy [19], and a comprehensive review synthesizes the evidence base [20]. Mechanistic studies provide biological plausibility for rapid analgesia via modulation of nociceptive transmission and neuroinflammation near the dorsal root ganglion [21-23]. Contemporary studies report predictors and short‑term outcomes across varied settings [24-29].

## Materials and methods

A prospective observational study was undertaken after obtaining approval from the Institutional Ethics Committee of Sri Devaraj Urs Medical College (approval number: SDUMC/KLR/IEC/306/2022-23) on a cohort of 35 consecutive patients who were presented to the orthopaedics department of RL Jalappa Hospital in Kolar, diagnosed as PIVD with LR.

The patient's comprehensive medical history, clinical examination, and radiological evaluation were documented. Written informed consent was obtained from the patients who were part of our study, fulfilling the inclusion and exclusion criteria.

Pain intensity was measured using the 11‑point Numeric Rating Scale (NRS) [30]. Disability was measured using the Oswestry Disability Index version 2.1a (ODI), scored per published guidance [31,32]. The ODI is used from Mapi Research Trust/ePROVIDE [33]. Neurodynamic status was assessed using the Straight‑Leg Raise Test (SLRT); its diagnostic utility and reliability have been summarized in systematic reviews [34,35].

Inclusion and exclusion criteria

The inclusion criteria consisted of patients aged 18 to 60 years who presented with radicular discomfort. Eligible patients were those whose symptoms persisted for more than one month and were not relieved by conservative management, who demonstrated an indication of PIVD on the corresponding nerve root as confirmed by lumbosacral MRI, and who were willing to provide written informed consent for the intervention. Patients were excluded if they presented primarily with back pain rather than radiating pain, had PIVD with spinal instability, a history of previous spinal surgeries, uncontrolled diabetes mellitus, prior adverse reactions to local anesthetics, or skin disorders at the injection site. Patients with cauda equina syndrome were also excluded.

Protocol

The patient was put in a prone position. Aseptic precautions were observed, parts were painted and draped, and a local anesthetic was injected into the skin and subcutaneous tissue at the area of interest by the orthopedic surgeon specialized in spine. A 22-gauge spinal needle was inserted into the pars interarticularis through the safety triangle (above and to the side of the departing nerve root). The location of the needle was verified under fluoroscopic C-ARM by capturing anteroposterior and lateral views. One milliliter of triamcinolone acetonide with two milliliters of bupivacaine was injected into each afflicted nerve root once the injection site had been confirmed. After the procedure, every patient was evaluated for the functional outcome using the ODI, NRS, and SLRT.

Statistical analysis

All data were collected, and statistical analysis was carried out using SPSS Statistics version 23.0 (IBM Corp., Released 2015. IBM SPSS Statistics for Windows, Version 23.0. Armonk, NY: IBM Corp.). Descriptive statistics for discrete variables were utilized to characterize the data through the implementation of frequency analysis and percentage analysis. Statistically significant differences between discrete variables in the two groups were examined using Fisher's exact test. The paired t-test was used to determine if there was a statistically significant change between the pre- and post-intervention values of continuous variables with a statistical significance level of 0.05.

## Results

The average age of participants was 39.63 ± 9.7 years, with 60% being males. The majority of the participants were farmers (31.2%), followed by drivers (22.9%), and 17.1% were clerks and teachers. The average duration of symptoms was 7.71 ± 0.926 months. The central region exhibited the highest frequency of herniation anatomical sites (57.1%), followed by the paracentral (42.9%).

We also noted the most commonly affected PIVD to be L4 and L5 (45.7%), followed by L3-L4 (28.6%) (Table 1). Lactate dehydrogenase (LDH) was most frequently observed at levels L4-L5 (35.7%), followed by L3-L4 (28.6%), with more radiating pain on the left side (57.1%). Bulge (57.1%) emerged as the prevalent morphology of the displaced disc material, with protrusion (31.4%) ranking as the second most frequent morphology.

**Table 1:** Representation of enrolled patients based on level of PIVD PIVD: prolapsed intervertebral disc

Level of PIVD	Frequency	Percent
L3-L4	10	28.6
L4-L5	16	45.7
L5-S1	9	25.7
Total	35	100

SNRB injection was performed most frequently at level L4 (35.7%), followed by level L5 (34.3%). The mean NRS score at preoperative, day 0, day 30, and day 90 was found to be 6.4, 3.29, 4.14, and 3.14, respectively, showing a drop in pain score after SNRB injection, which was statistically significant. Further, the mean ODI score at preoperative, day 0, day 30, and day 90 was found to be 14.31, 7.83, 7.86, and 6.86, respectively, which also showed a decline after SNRB injection.

After SNRB injection at day 0, all the patients tested SLRT negative, and it remained negative even after 30 days. Ninety days after SNRB injection, only four patients tested SLRT positive, and it was statistically significant by Fisher's exact test (p = 0.0001) (Table 2).

**Table 2:** Comparison of SLRT before and after intervention Fisher’s exact value = 54.84 SLRT: Straight‑Leg Raise Test

SLRT	Preoperative	Postoperative day 0	30 days	90 days	p-value
Positive	35	0	0	4	0.0001
Negative	0	35	35	31	

The mean NRS score decreased significantly (paired t-test) at each of the following three months and postoperative days compared to the preoperative mean score (0, 1, and 90 days). The mean NRS score exhibited a statistically significant increase at one month against the postoperative day 0 score, followed by a statistically insignificant decrease at three months. In LR associated with PIVD, SNRB injections continue to ameliorate pain three months after administration; however, pain levels marginally increase one month after the injection date (Table 3).

**Table 3:** Assessment of NRS score before and after SNRB injection by paired t-test NRS: Numeric Rating Scale, SNRB: selective nerve root block, SD: standard deviation

Pairs	Assessment of NRS score before and after SNRB injection	Mean ± SD	Mean difference	p-value
Pair 1	Preoperative at baseline	6.40 ± 0.78	3.114	0.0001
Pair 1	Postoperative on day 0	3.29 ± 0.57	3.114	0.0001
Pair 2	Preoperative at baseline	6.40 ± 0.78	2.257	0.0001
Pair 2	Postoperative at 30 days	4.14 ± 0.97	2.257	0.0001
Pair 3	Preoperative at baseline	6.40 ± 0.78	3.257	0.0001
Pair 3	Postoperative at 90 days	3.14 ± 0.55	3.257	0.0001
Pair 4	Postoperative on day 0	3.29 ± 0.57	-0.857	0.0001
Pair 4	Postoperative at 30 days	4.14 ± 0.97	-0.857	0.0001
Pair 5	Postoperative on day 0	3.29 ± 0.57	0.143	0.201
Pair 5	Postoperative at 90 days	3.14 ± 0.55	0.143	0.201

The postoperative mean ODI score decreased significantly (paired t-test) at 0 days, 30 days, and 90 days in comparison to the preoperative mean ODI score. This decrease was statistically significant. When comparing the mean ODI score to the score on postoperative day zero, there is no statistically significant difference observed at one month. However, a significant decrease is observed at three months. As a result, SNRB injections continue to reduce the degree of disability in patients with LR and PIVD even three months after administration; however, at one month, the degree of disability remains unchanged from the day of administration (Table 4).

**Table 4:** Assessment of ODI score before and after SNRB injection by paired t-test ODI: Oswestry Disability Index, SNRB: selective nerve root block, SD: standard deviation

Pairs	Assessment of the ODI score before and after SNRB injection	Mean ± SD	Mean difference	p-value
Pair 1	Preoperative at baseline	14.31 ± 3.43	6.486	0.0001
Pair 1	Postoperative on day 0	7.83 ± 2.26	6.486	0.0001
Pair 2	Preoperative at baseline	14.31 ± 3.43	6.457	0.0001
Pair 2	Postoperative at 30 days	7.86 ± 2.68	6.457	0.0001
Pair 3	Preoperative at baseline	14.31 ± 3.43	7.457	0.0001
Pair 3	Postoperative at 90 days	6.86 ± 2.39	7.457	0.0001
Pair 4	Postoperative on day 0	7.83 ± 2.26	-0.029	0.922
Pair 4	Postoperative at 30 days	7.86 ± 2.68	-0.029	0.922
Pair 5	Postoperative on day 0	7.83 ± 2.26	0.971	0.0001
Pair 5	Postoperative at 90 days	6.86 ± 2.39	0.971	0.0001

## Discussion

In this prospective cohort, fluoroscopy‑guided SNRB for single‑level disc‑related LR was associated with rapid and durable improvements in pain, disability, and neurodynamic signs over 90 days. Mean NRS declined from 6.40 at baseline to 3.29 immediately, 4.14 at 30 days, and 3.14 at 90 days; ODI improved from 14.31 to 7.83, 7.86, and 6.86, respectively. SLRT converted from positive pre‑procedure to negative at day 0 and day 30 in all patients, with 4/35 (11.4%) positive at day 90. These effect sizes are consistent with contemporary cohorts using transformational techniques [19,26-28] and with a comprehensive review [20].

Mechanistically, periradicular corticosteroid near the dorsal root ganglion can suppress nociceptive C‑fiber transmission, attenuate local neuroinflammation, and limit sympathetic sprouting mechanisms aligned with the rapid analgesic response observed after SNRB [21-23]. Diagnostic blocks also aid localization when imaging and clinical findings are discordant [13,15].

Our findings fit within a broader literature indicating short‑term effectiveness of SNRB or related transforaminal injections for disc‑related radiculopathy [24,25,27-29]. Systematic reviews summarize SLRT’s diagnostic utility and reliability in radicular syndromes [34,35], supporting its use alongside patient‑reported outcomes to track recovery.

LR is less prevalent than LBP alone and can vary from 9% to 25%. Back pain radiating into the legs, foot, and toes is the hallmark of LR [18]. Although 23% to 48% of patients get a spontaneous resolution of their symptoms after a year, 30% may continue to experience severe symptoms, 20% may lose their jobs, and 5% to 15% may need surgery for their condition. LR is caused by a prolapsed disc [19].

There are a variety of therapeutic approaches for LR because the condition is caused by numerous pathophysiological causes [20]. One of the methods is injecting steroids into the SNRB, as they have a therapeutic impact because of their ability to stabilize nociceptive signals and reduce inflammation [21-23]. Many studies have shown that SNRB is effective, with results ranging from 76% to 88% [24].

The mean age of the enrolled patients in this study was 39.63 ± 9.7 years, ranging from 23 to 60 years, respectively, with a definite male predilection, with 60% being males and the remaining 40% being females. Similarly, Vashishtha et al. [25] reported a mean age of 39.68 ± 8.85 years, with a gender ratio of 2.33:1. Dhandapani et al. [26] reported a mean age of 43.22 ± 9.97 years. The enrolled patients consisted of 27 males (51.92%) and 25 females (48.07%). Khan et al. [19] found an average age of 38.6 with 65.83% men and 34.31% women.

The mean spell of symptoms of the enrolled patients in this study was 7.71 ± 0.926 months, with the most common side of radiating pain being the left side (57.1%), followed by the right side (42.9%). In 2015, Khan et al. [19] examined 122 patients in India who had LDH, pain in their backs, and LR. The patients were seen to have failed to show improvement following six weeks of medical therapy. Seventeen patients (34%) [25] reported discomfort in the right side, compared to 15 patients (30%), whereas 17 patients (34%) reported discomfort in the left side.

In the current study, 45.7% of participants had PIVD at level L4-L5, with levels L3-L4 coming in second at 28.6%. The most common level of SNRB injection is L4 (45.7%), followed by L5 (34.3%), with the anatomical location of the herniation being central (57.1%) and paracentral (42.9%).

Nevertheless, Khan et al. [19] found that after six weeks of conservative treatment, the patients still did not show any improvement amongst 56 (46.67%) patients with prolapsed discs at the L4-L5 level and 39 (32.50%) at the L5-S1 level. Kanaan et al. [27] found that 32 individuals had LDH in the L4-L5 region, whereas Vashishtha et al. [25] reported that three-quarters of patients had abnormalities in the L4-L5 intervertebral disc, 20% in the L5-S1 disc, and the largest percentage in the L4-L5-S1 disc. Dhandapani et al. [26] reported that 31 patients (59.6% of the total) had pathologies at the L4-5 level, 10 patients (19.2%) had abnormalities at the L5-S1 level, and 11 patients (21.1%) had problems at the level beyond those two levels.

All our patients tested positive with the SLRT before SNRB injection and after SNRB injection at day 0, tested negative with the SLRT, and remained negative in the next 30 days. At the end of 90 days after SNRB injection, only four patients tested SLRT positive, and it was statistically significant by Fisher’s exact test (p = 0.0001). Khan et al. [19] studied 120 patients who reported that even after six weeks of conservative treatment, the patients still did not show any improvement, wherein average SLRT grew from 43.42 ± 10.99 at the beginning to 67.78 ± 6.23 following six months of follow-up.

We found that the pain score decreased immediately after SNRB injection at day 0 of intervention (3.29). However, this pain score increased at 30 days (4.14), and later it decreased at 90 days after intervention (3.14). In comparison to the preoperative mean NRS score, the mean NRS score declined postoperatively on day 0, 30 days, and 90 days, displaying a drop in mean NRS score, which was found to be statistically significant (Table 3). Thus, SNRB injection helps to alleviate the pain even 90 days after injection in LR in PIVD, but there is a slight increase in pain at 30 days compared to the day of injection. Patients did not require surgery since the local anesthetic agent provided immediate pain relief and continued to do so for three months.

A similar drop in NRS score was observed in the studies carried out by Shaikh et al. [28], Khan et al. [19], Singh et al. [29], Vashishtha et al. [25], and Dhandapani et al. [26]. The disability assessment score decreased immediately after SNRB injection at day 0 of intervention (7.83). However, this disability score remained the same at 30 days (7.86), and later it decreased at 90 days after intervention (6.86).

In comparison to the preoperative mean ODI score, the mean ODI score was reduced at postoperative day 0, 30 days, and 90 days, and this decrease in mean ODI score was statistically significant by paired t-test (Table 4). Therefore, SNRB injection helps to reduce the degree of disability even 90 days after injection in LR in PIVD, but there is no difference in the degree of disability at 30 days compared to the day of injection.

Shaikh et al. [28], who monitored SNRBs for three months, reported that three weeks and three months after injection, the ODI scores of 42 patients who opted out of surgery dropped dramatically from 59.4 (SD 14.69) before injection to 42.4 (SD 9.33), 38.6 (SD 11.99), and 26.3 (SD 9.43) (p < 0.001), respectively. Khan et al. [19] reported a baseline score on the ODI was 82.10 ± 3.8. However, after three months, the score dropped to 38.6 ± 6.10, indicating a significant reduction.

Another RCT study by Singh et al. [29] reported the ODI score at day 0 to be 78.20 ± 2.8, which dropped to 39.55 ± 5.10 after 90 days. Vashishtha et al. [25] also reported an ODI score of 16.56 ± 10.3 on the 90th day in comparison to a score of 49.88 ± 17.18 on day 0.

We found a continuous decline across all assessment scores for a postoperative period of 90 days, reflecting the efficacy of SNRB. However, we could not carry out an extensive postoperative follow-up beyond that. Therefore, we advocate similar studies to understand the effectiveness of SNRB while providing more data concerning the predictive treatment outcome.

Clinical implications

Clinical implications include the ability to deliver rapid analgesia that facilitates early activity and rehabilitation, as well as providing a diagnostic adjunct to confirm the symptomatic level when imaging and examination are discordant. Such interventions may also help reduce short-term disability while definitive management plans are being formulated and can assist in identifying non-responders within 4 to 12 weeks who may require alternative interventions. These points are most applicable when clinical and imaging findings are concordant and when timely pain control can prevent deconditioning [13,16,20,24-29].

Limitations

This study has several limitations. First, it is a single‑center observational cohort with a modest sample size (n = 35), which limits generalizability and precludes robust subgroup analyses by level, laterality, or disc morphology. Second, outcomes were assessed over a short horizon (immediate, 30 days, and 90 days); longer follow‑up is required to ascertain durability and late conversions to surgery. Third, without a concurrent comparator (e.g., sham injection or alternative epidural approach), expectancy effects or regression to the mean cannot be excluded, although the magnitude of paired changes argues for a true treatment effect. Finally, we did not stratify by injectate composition or volume, which can influence response.

## Conclusions

Administration of SNRB injection effectively decreases the intensity of pain and level of impairment in individuals with LR caused by PIVD, even up to 90 days following the injection. SNRB is an easy, cost-effective, and safe way to alleviate pain both temporarily and over the long term, and it also improves functional outcomes. This procedure is a quick and efficient intervention that may be done as an outpatient procedure, without the need for anesthesia. Thus, the use of SNRB provides both diagnostic and therapeutic advantages in the management of patients. Therefore, we suggest using a nerve root block as a first measure before resorting to surgical intervention for patients with LR caused by PIVD.
